# Physical environment as a catalyst for healthy aging: servicescape, motivation, and well-being in older adult park golf

**DOI:** 10.3389/fspor.2026.1892286

**Published:** 2026-08-13

**Authors:** Jaepil Seo, Kisun Hwang, Joonha Shin, Seung-Hwan Woo, Young-Kyun Sim

**Affiliations:** 1 Sports & Leisure Studies, Gimcheon University, Gimcheon-si, Gyeongsangbuk-do, Republic of Korea; 2 Department of Recreation and Leisure Sports, Dankook University, Cheonan-si, Chungcheongnam-do, Republic of Korea; 3 Department of Sports in Life, Jangan University, Hwaseong-si, Gyeonggi-do, Republic of Korea; 4 Department of Physical Education, Woosuk University, Wanju-gun, Jeollabuk-do, Republic of Korea; 5 Department of Sports Welfare Education, Woosuk University, Wanju-gun, Jeollabuk-do, Republic of Korea

**Keywords:** aging society, happiness, health, older adult activity, successful aging

## Abstract

**Background/objective:**

Active leisure activities like park golf are crucial for older adults' successful aging. Grounded in the Stimulus-Organism-Response (S-O-R) paradigm and Self-Determination Theory (SDT), this study investigated the structural relationships among perceived servicescape, facility image, satisfaction, motivation, and subjective well-being.

**Methods:**

A quantitative cross-sectional design was employed. Data from 264 older adult park golf participants in South Korea were analyzed using structural equation modeling. A phantom variable approach was employed to rigorously test multiple mediation effects.

**Results:**

The physical servicescape (stimulus) positively impacted both perceived image and facility satisfaction (organism), which subsequently enhanced participation motivation (response). The structural model showed an acceptable fit (*χ*^2^/*df* = 1.831, TLI = .904, CFI = .919, RMSEA = .092, SRMR = .074). Servicescape significantly predicted both image (*β* = .838, *p* < .001) and satisfaction (*β* = .699, *p* < .001), and image (*β* = .447, *p* < .001) and satisfaction (*β* = .299, *p* < .01) in turn predicted motivation, which strongly predicted subjective well-being (*β* = .774, *p* < .001). Notably, perceived symbolic image influenced motivation more strongly than functional satisfaction. This voluntarily formed motivation then was associated with higher levels of participants' subjective well-being.

**Conclusion:**

Beyond establishing excellent physical infrastructure, fostering an emotionally appealing facility image is vital for promoting continuous sports participation and genuine psychological well-being among older adults. These findings provide empirical evidence for infrastructure design and emotion-oriented marketing in age-friendly sports facilities. Ultimately, this research offers practical insights into utilizing leisure environments as public health interventions to promote successful aging.

## 1 Introduction

Modern society is experiencing rapid population aging due to advancements in medical technology, and the World Health Organization (1) projects that the population aged 60 and older will account for one-sixth of the global population by 2050. The deterioration of physical and mental health among older adults directly leads to restrictions in daily life and a substantial increase in social costs. Consequently, academia continuously emphasizes the importance of active leisure activities for “successful aging”—which goes beyond merely extending lifespan to living a healthy, disease-free old age (2, 3).

Amidst this trend, park golf, which combines regular walking with social interaction, has recently gained attention as an alternative sport among older adults in South Korea (4). Park golf is a physical activity that imposes minimal physical strain, is highly accessible, and is economical, allowing older adults to participate continuously without the risk of injury (5). Furthermore, it is evaluated as a positive model for lifetime sports, as navigating the course and communicating with companions helps prevent social isolation and contributes to the formation of social capital in later life (6). Regular participation in physical activities such as park golf ultimately serves as a significant factor in enhancing the subjective well-being of older adults (7). Subjective well-being is a multidimensional concept encompassing life satisfaction, which is a cognitive evaluation of one's own life, as well as the positive and negative affects experienced in daily life (8). Regular outdoor exercise activates physiological and psychological processes that promote the secretion of positive brain hormones, thereby increasing positive affect and effectively alleviating negative affects such as depression in old age (9).

However, participation in physical activities does not always guarantee the same level of benefits; rather, it depends significantly on the qualitative level of participation motivation (10). According to Self-Determination Theory (SDT), the intrinsic and extrinsic motivations of individuals participating in sports influence the promotion of their well-being (11, 12). For instance, a recent study focusing on sports participants with disabilities (13) reported that self-determined motivation, comprising both intrinsic and extrinsic factors, has a significant impact on participants' life satisfaction and positive affect. Consequently, it can be inferred that the motivation levels of older adult sports participants are also a prerequisite for preventing dropout and providing psychological benefits such as well-being (14, 15).

The motivational formation process that leads older adult participants to continuously immerse themselves in park golf can be explained by the Stimulus-Organism-Response (S-O-R) paradigm (16). The S-O-R model, a multidimensional behavioral framework, posits that external physical and environmental stimuli alter an organism's internal cognitive and emotional states, ultimately eliciting specific behavioral responses. Because park golf takes place in outdoor park environments, the comfort and physical infrastructure of the sports facilities serve as the dominant initial stimuli for participants. “Servicescape,” a concept that materializes this physical environment, encompasses all tangible elements of the space where the service is provided, such as rational spatial layout, natural landscaping, and the comfort of amenities (17). A systematically managed servicescape provides psychological stability to older adults who may have diminished visual cognitive abilities, acting as environmental capital that may influence continuous facility usage and sports immersion (17–19).

Participants continuously exposed to an excellent servicescape (stimulus) form two internal organismic states regarding the target space: a “perceived image” and service “satisfaction”. The perceived image of a park golf course refers to the holistic and favorable cognitive and emotional representation that participants build in their minds as they experience the facilities and environment (20). A comfortable facility environment stimulates intrinsic motivation by imprinting a positive image of the space as a safe, friendly, and age-friendly area (21). Furthermore, based on expectancy-disconfirmation theory, satisfaction is an emotional evaluation that occurs when the quality of the actual service experience meets or exceeds prior expectations (22). A pleasant environment in a sports facility induces a level of satisfaction that surpasses customer expectations, acting as a core cognitive response that multiplies autonomous participation intent and immersion (23, 24).

The cognitive organismic state, having imprinted a favorable image and experienced high service satisfaction based on environmental stimuli, ultimately transitions into the visible behavioral response of “exercise motivation”. Older adults who enjoy psychological benefits while using safe and systematically managed park golf courses internalize intrinsic motivations for the enjoyment of the physical activity itself, skill acquisition, and socialization (25). As previously discussed in the context of SDT, firmly established voluntary sports participation motivation alleviates the depression frequently encountered in old age and instills vitality into life. Consequently, this motivation serves as a key catalyst in enhancing the subjective well-being of older adults. In short, a logical, structural causal flow is derived wherein the physical environment—the servicescape—induces motivation mediated by perceived image and satisfaction, which in turn leads to improved well-being.

Despite the importance of logic integrating the S-O-R paradigm and SDT, previous studies related to park golf present several limitations: First, the majority of research has been confined to identifying unidirectional, one-dimensional relationships between fragmented variables, such as the relationship between participation motivation and facility satisfaction. Second, there has been a lack of attempts to organically verify, within a structural model, the multi-stage trajectory in which physical environmental factors lead to psychological well-being through internal responses. Third, from a statistical methodology perspective, there has been a limitation of reporting only the total indirect effect without strictly separating and independently verifying the significance of the “specific indirect effects” that multiple mediators exert on the dependent variable (26). Finally, compared to the social value of park golf, there remains a shortage of large-scale empirical data to quantitatively support it.

Therefore, this study aims to conduct systematic structural model verification to address the limitations of existing research. Specifically, targeting older adult park golf participants, this study seeks to verify the process by which the servicescape promotes participation motivation and ultimately leads to subjective well-being, with perceived image and satisfaction acting as multiple mediators. This study will provide empirical evidence for establishing infrastructure design standards and efficient service marketing strategies for sports facilities targeting older adults in the future. Furthermore, through multidisciplinary integration, it aims to contribute to improving the quality of life and successful aging of older adults in the approaching super-aged society.

To this end, the research questions established in this study are as follows: Research Question 1, What is the overall structural causal relationship among the servicescape, perceived image, satisfaction, motivation, and subjective well-being perceived by older adult park golf participants? Research Question 2, Do perceived image and satisfaction each play a distinct and significant mediating role in the pathway between the servicescape and motivation?

## 2 Materials and methods

### 2.1 Participants

A total of 264 older adults who regularly participate in park golf in South Korea participated in this study. Sample size adequacy was verified using G*Power 3.1. For a linear multiple regression with five predictors, a medium effect size (f^2^ = 0.15), alpha = .05, and.95 power, the minimum required sample was 138. The final sample of 264 exceeds both this calculated minimum and the recommended threshold for structural equation modeling (27). Detailed procedures for participant selection are described in the Procedures section.

### 2.2 Measures

#### 2.2.1 Servicescape

The servicescape was measured using a questionnaire developed for park golf course users in South Korea (28). This instrument consists of 20 items across four factors perceived by park golf participants: space arrangement (6 items), location condition (5 items), environmental condition (3 items), and course management (6 items). Each item was rated on a 5-point Likert scale ranging from 1 (strongly disagree) to 5 (strongly agree). In this study, the internal consistency coefficients (Cronbach's *α*) were.797 for space arrangement,.743 for location condition,.828 for environmental condition, and.845 for course management.

#### 2.2.2 Image of the park golf course

To measure participants' perceived image of the park golf course, a Korean version of the questionnaire (29) based on Baloglu and McCleary's (20) “model of destination image formation” was utilized. The term “sports center” in the original items was modified to “park golf course,” and content validity was reviewed. The image of the park golf course comprises 8 items across two factors: emotional image (5 items) and functional image (3 items). Each item was measured on a 5-point Likert scale ranging from 1 (strongly disagree) to 5 (strongly agree). The internal consistency coefficients in this study were.940 for emotional image and.920 for functional image.

#### 2.2.3 Participation satisfaction

Participants' satisfaction was assessed using a questionnaire (30) that reflects South Korea's cultural characteristics, adapted from a satisfaction scale for sports and leisure facilities developed overseas (31). This scale consists of 11 items across three factors: satisfaction with facilities (4 items), satisfaction with programs (4 items), and satisfaction with services (3 items). Each item was rated on a 5-point Likert scale ranging from 1 (strongly disagree) to 5 (strongly agree). The internal consistency coefficients for this study were.815 for facility satisfaction,.869 for program satisfaction, and.919 for service satisfaction.

#### 2.2.4 Motivation

The motivation of park golf participants was measured using the Korean version (32) of The Sport Motivation Scale (SMS; 33) originally developed overseas. This scale measures intrinsic motivation, extrinsic motivation, and amotivation regarding sports participation. Specifically, it consists of 4 items for enjoyment, 7 items for skill development and achievement, 5 items for health and fitness, 4 items for socialization, 4 items for extrinsic display, and 5 items for amotivation. However, the amotivation factor was excluded in this study as it did not align with the research purpose, resulting in a motivation measure of 24 items across 5 factors. Each item was rated on a 5-point Likert scale ranging from 1 (strongly disagree) to 5 (strongly agree). The internal consistency coefficients in this study were.955 for enjoyment,.955 for skill development and achievement,.884 for health and fitness,.892 for socialization, and.919 for extrinsic display.

#### 2.2.5 Subjective well-being

The subjective well-being of the participants was assessed using two distinct scales. First, life satisfaction was measured using the Korean version (34) of the Satisfaction With Life Scale (SWLS; 8). This scale has a single-factor structure with a total of 5 items, each rated on a 5-point Likert scale ranging from 1 (strongly disagree) to 5 (strongly agree). In this study, the internal consistency coefficient was.929. Meanwhile, to measure positive and negative affects for subjective well-being, the Korean version (35) of the Positive and Negative Affect Schedule (PANAS; 36) was utilized. This scale includes a total of 20 items: 10 for positive affect and 10 for negative affect. Each item was rated on a 5-point Likert scale ranging from 1 (strongly disagree) to 5 (strongly agree). The internal consistency coefficients in this study were.964 for positive affect and.973 for negative affect.

### 2.3 Procedures

Prior to the data collection process, official preliminary review and approval were obtained from the Institutional Review Board (IRB) to strictly ensure ethical validity and fully protect the human rights and personal information of the participants. Subsequently, surveys were conducted by directly visiting approximately 10 large-scale public and private park golf courses operating in various regions of South Korea. Data were collected from May to July in 2025. To secure the representativeness of the sample while considering the practical constraints of data collection, participants were selected using a reasonable combination of purposive sampling, which accounted for regional distribution, and convenience sampling, which considered the accessibility of on-site visitors. Participants were eligible for inclusion if they (a) were aged 60 years or older, (b) had been participating in park golf on a regular basis (participating in park golf for at least 3 months), and (c) voluntarily provided written informed consent after being informed of the purpose of the study. Respondents were excluded if they (a) provided careless or internally inconsistent responses, (b) had an excessive number of missing values, or (c) were younger than 60 years of age.

To maximize the statistical reliability of the collected data and the quality of the responses, five trained surveyors (including the researchers), who had completed specialized prior training on survey methodology, were dispatched directly to the park golf courses. They strategically utilized the participants' free waiting time before starting a round or resting times between holes to kindly explain the purpose of the study, its academic intent, and the strict guarantee of anonymity for the responses in detail. All questionnaires were distributed only after the participants fully understood the research intent and confirmed their voluntary participation through written informed consent. The survey was self-administered; however, for some participants who experienced decreased vision due to old age or complained of difficulties in reading the items and writing directly, the surveyors provided assistance. A total of 300 questionnaires were initially distributed to the participants, of which 285 were retrieved (a recovery rate of 95.0%). Following a rigorous data cleaning process, 12 responses were excluded due to significantly inconsistent response patterns or excessive missing values. In addition, 9 respondents younger than 60 years of age were excluded to ensure that the sample reflected the older adult population targeted in this study. Consequently, a final total of 264 valid samples were utilized for formal hypothesis testing and statistical analysis.

### 2.4 Analysis

To objectively and scientifically verify the hypotheses and theoretical structural model established in this study, the collected quantitative data were analyzed from multiple angles using the IBM SPSS Statistics 27.0 and AMOS 27.0 software packages. The overall analysis procedure was conducted through highly systematic statistical steps. First, a frequency analysis was conducted to identify the socio-demographic characteristics and park golf participation patterns of the respondents. Additionally, to thoroughly check the overall tendencies, concentration, and multivariate normality assumptions of the main measurement variables constituting this study, descriptive statistics were calculated, including the mean, standard deviation, skewness, and kurtosis. To assess the potential threat of common method bias arising from the self-reported nature of the data, Harman's single-factor test was conducted prior to the main analyses. Second, to rigorously verify the reliability of the items comprising the measurement tools, Cronbach's *α* was calculated to confirm internal consistency. Furthermore, Pearson's product-moment correlation analysis was conducted to closely examine the occurrence of multicollinearity and the direction of correlations among the variables.

Third, a Confirmatory Factor Analysis (CFA) using Maximum Likelihood (ML) was conducted beforehand to verify the construct validity of the measurement tools, confirming whether the measured variables appropriately explained the latent variables according to the theoretical structure. The overall goodness-of-fit of the measurement model was comprehensively evaluated by cross-considering various fit indices, including *x*^2^/*df*, Tucker–Lewis Index (TLI), Comparative Fit Index (CFI), Root Mean Square Error of Approximation (RMSEA), and Standardized Root Mean Square Residual (SRMR), taking the sensitivity of the data into account. Fourth, to verify the directional hypotheses of the proposed research model and the hypothesized relationships among the latent variables, Structural Equation Modeling (SEM) analysis was officially conducted. The goodness-of-fit of the structural model was strictly evaluated through the same system of fit indices used in the CFA (*x*^2^/*df*, TLI, CFI, RMSEA, and SRMR).

Fifth, as the most essential procedure of this study's analysis, the bootstrapping technique was utilized to verify the statistical significance of the indirect effect of the independent variable, servicescape, on the dependent variable, participation motivation, through the multiple mediators of perceived image and satisfaction. To verify the significance of the mediating effect, this analysis involved 2,000 random resamplings from the original data to establish a Bias-Corrected 95% Confidence Interval, and the mediation effect was determined by strictly checking whether zero was included between the upper and lower limits. Notably, the structural model of this study takes the form of a multiple mediation model where two mediating variables exist in parallel. To verify this, the phantom variables approach (26) was applied to the analysis model. A phantom variable is an analytical, virtual latent variable without actual data values. It is an advanced method in which a virtual path model with mathematically identical parameter constraints to the specific indirect path of interest (e.g., servicescape, image, motivation) is additionally designed and connected within the AMOS structural equation visualization screen. In this analysis, virtual variables were set, and the mediation significance of the two independent indirect effects was individually verified through bootstrapping confidence intervals.

## 3 Results

### 3.1 Participant characteristics

Of the 264 older adult participants, 162 were male (61.4%) and 102 were female (38.6%). Their ages ranged from 60 to 77 years (*M* = 65.85, *SD* = 5.05). The duration of park golf participation ranged from 1 month to 13 years (*M* = 2.31 years, *SD* = 2.22), and participants played park golf on average 3.14 times per week (*SD* = 1.47): five or more times (*n* = 72, 27.3%), twice (*n* = 54, 20.5%), once (*n* = 48, 18.2%), three times (*n* = 48, 18.2%), and four times (*n* = 42, 15.9%) per week. The demographic characteristics of the participants are presented in Table 1.

**Table 1:** Demographic characteristics of participants (*N* = 264).

Categories	Subcategories	N	%
Gender	Male	162	61.4
	Female	102	38.6
Education	Middle school	18	6.8
	High school	48	18.2
	University	141	53.4
	Graduate school	48	18.2
	Missing	9	3.4
Weekly frequency	Once	48	18.2
	Twice	54	20.5
	3 times	48	18.2
	4 times	42	15.9
	More than 5 times	72	27.3
Park golf experience	Less than 2 years	120	45.5
	2 to 3 years	99	37.5
	More than 3 years	45	17.0
Total		264	100.0

### 3.2 Descriptive statistics and correlations

Regarding common method bias, Harman's single-factor test showed that the first unrotated factor accounted for 34.748% of the total variance, which is below the 50% criterion. This indicates that common method bias was not a serious concern in this study.

Table 2 presents the descriptive statistics and correlations of the main variables used in this study. The means of all variables ranged from 1.74 (negative affect) to 3.89 (functional image), and the standard deviations were between 0.75 and 1.08, showing an overall appropriate level of variability. Skewness ranged from −1.09 to 1.39, and kurtosis ranged from −0.46 to 3.12; as most variables fell within an absolute value of 2, the assumption of normality was considered met. Significant positive correlations were found among the servicescape factors (*r* = .588–.745, *p* < .01), suggesting that the components of the physical environment are closely interrelated. The emotional and functional images comprising the park golf course image showed a high correlation (*r* = .842, *p* < .01) and exhibited moderate-to-high positive correlations with the servicescape factors (*r* = .443-.654, *p* < .01). Customer satisfaction also demonstrated strong associations among facility, program, and service components (*r* = .631–.782, *p* < .01), and consistently showed significant positive correlations with the servicescape and image factors. The sub-factors of exercise participation motivation displayed high correlations with one another (*r* = .482–.837, *p* < .01) and generally exhibited positive correlations with customer satisfaction and image factors. Finally, regarding subjective well-being, life satisfaction and positive affect were positively correlated with most of the main variables (*r* = .222–.627, *p* < .01), whereas negative affect showed only weak negative correlations with some variables. Overall, the correlations among the main constructs of this study were confirmed in the theoretically expected directions.

**Table 2:** Mean, standard deviation, skewness, kurtosis, and correlation coefficients of observed variables.

Factors	1	2	3	4	5	6	7	8	9	10	11	12	13	14	15	16	17
Space arrangement	1																
Course management	.745**	1															
Location condition	.592**	.708**	1														
Environmental condition	.588**	.620**	.593**	1													
Emotional image	.576**	.649**	.650**	.637**	1												
Functional image	.443**	.569**	.647**	.654**	.842**	1											
Satisfaction with facilities	.557**	.551**	.448**	.553**	.604**	.517**	1										
Satisfaction with programs	.514**	.592**	.550**	.504**	.567**	.556**	.631**	1									
Satisfaction with services	.500**	.593**	.492**	.443**	.559**	.513**	.665**	.782**	1								
Enjoyment	.357**	.338**	.388**	.452**	.550**	.568**	.623**	.378**	.340**	1							
Skill development and achievement	.389**	.419**	.526**	.515**	.534**	.595**	.571**	.478**	.444**	.777**	1						
Health and fitness	.402**	.400**	.519**	.493**	.546**	.610**	.581**	.516**	.440**	.813**	.837**	1					
Socialization	.271**	.284**	.395**	.313**	.413**	.492**	.342**	.315**	.305**	.561**	.577**	.670**	1				
Extrinsic display	.411**	.333**	.441**	.376**	.332**	.377**	.375**	.419**	.382**	.482**	.666**	.646**	.609**	1			
Satisfaction with life	.278**	.321**	.324**	.382**	.485**	.521**	.488**	.379**	.341**	.583**	.566**	.619**	.547**	.444**	1		
Positive affect	.228*	.263**	.281**	.186	.368**	.369**	.420**	.269**	.222*	.503**	.482**	.517**	.434**	.392**	.627**	1	
Negative affect	−.008	−.059	−.010	−.179	−.237*	−.204*	−.101	−.014	−.163	−.223*	−.089	−.177	−.146	.033	−.184	−.099	1
*M*	3.274	2.766	3.040	3.468	3.745	3.892	3.346	3.081	3.128	3.755	3.658	3.705	3.646	3.255	3.446	3.481	1.741
*SD*	.812	.871	.776	.921	.878	.880	.807	.908	1.078	.815	.839	.750	.791	.889	.815	.922	.926
Skewness	.120	.624	.212	−.342	−.520	−.635	−.530	−.142	−.424	−.784	−.621	−1.086	−.316	−.351	−.391	−.507	1.390
Kurtosis	−.392	.150	.831	−.019	.004	.159	.385	−.236	−.460	1.378	1.017	3.123	.738	.385	.619	.442	1.266

* *p* < .05.

** *p* < .01.

### 3.3 Structural equation model

**Figure 1:**
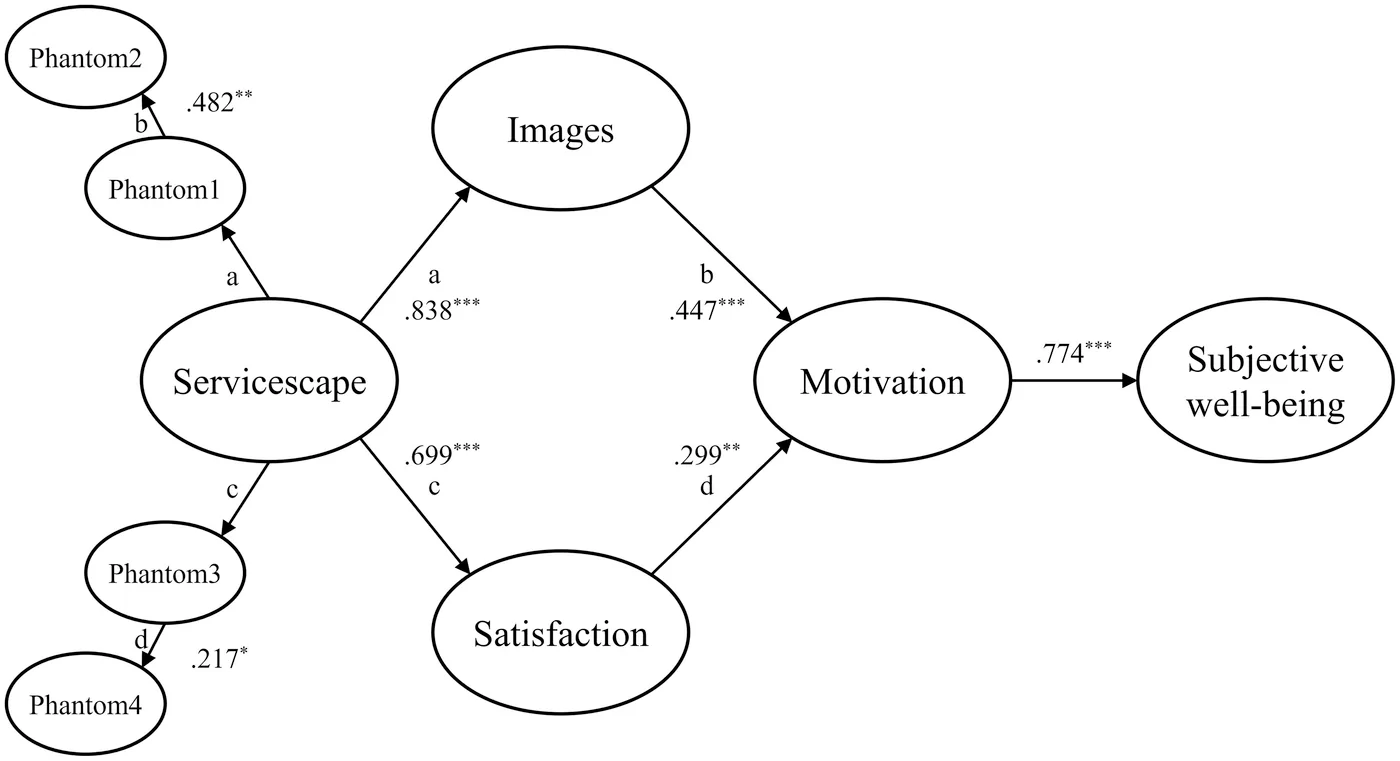
Tested model with phantom variables and standardized estimates.

Table 3 and Figure 1 provide the results of the structural equation modeling analysis. The model fit indices—*x*^2^  = 208.684, *df* = 114 (*x*^2^/*df* = 1.831), TLI = .904, CFI = .919, RMSEA = .092 [90% CI = (.072,.112)], SRMR = .074—indicated an acceptable fit to the data. Although the RMSEA slightly exceeded the conventional cutoff of.08, this index is known to be sensitive to model complexity and to penalize models with a small sample size, which can inflate its value even when the model is well specified (37). Given that the incremental fit indices (TLI = .904, CFI = .919) met the recommended threshold of .90, the SRMR (.074) satisfied the criterion of <.08, and the lower bound of the RMSEA 90% confidence interval (.072) fell below .08, the overall fit was judged acceptable for interpreting the structural relationships.

**Table 3:** Estimates and standardized estimates of all paths of research model.

Direct paths	Estimate	Std. Estimate	S.E.	*t*	*p*
Servicescape → Images	.993	.838	.129	7.689	***
Servicescape → Satisfaction	.945	.699	.158	5.996	***
Image → Motivation	.390	.447	.102	3.815	***
Satisfaction → Motivation	.229	.299	.089	2.581	.01
Motivation → Subjective well-being	.797	.774	.102	7.832	***

Indirect Paths	Estimate	S.E.	*p*	95% CI	
				Lower	Upper
Servicescape → Images → Motivation	.482	.131	.005	.144	.665
Servicescape → Satisfaction → Motivation	.217	.108	.023	.026	.462

*** *p* < .001.

The servicescape had significant positive effects on both image (*β* = .838, *p* < .001) and satisfaction (*β* = .699, *p* < .001). In addition, image significantly influenced motivation (*β* = .447, *p* < .001), and satisfaction also showed a significant positive effect on motivation (*β* = .299, *p* = .010). Participation motivation exhibited a strong positive effect on subjective well-being (*β* = .774, *p* < .001), suggesting that motivation plays a core mediating role in the well-being formation process. In the indirect effect analysis, the indirect effect of the servicescape on motivation through image was significant (B = .482, 95% CI = .144,.665, *p* < .01), and the indirect effect through satisfaction was also significant (B = .217, 95% CI = .026,.462, *p* < .05). The statistical significance of the mediating effects was confirmed, as the confidence intervals for both paths did not include zero. These results demonstrate that rather than directly affecting motivation, the servicescape exerts an indirect influence mediated by image and satisfaction.

## 4 Discussion

This study empirically verified the multidimensional and dynamic interrelated structure among the perceived servicescape of sports facilities, the complex image of park golf courses, overall satisfaction, motivation, and subjective well-being. This was conducted among 264 older adults participating in park golf, which has rapidly emerged as a representative lifetime sport for older adults in South Korea, a country on the verge of becoming a super-aged society, by combining the S-O-R paradigm and SDT. The verification indices of the structural model derived from the results were consistent with the theoretical foundation, and an in-depth, comprehensive academic interpretation of these findings can be unfolded as follows.

First, the servicescape of the facility, which corresponds to the initial stimulus in the S-O-R model, was found to have a strong positive effect on both the image of the park golf course and customer satisfaction, which represent the organismic state (*β* = .838, *β* = .699). This implies that visible physical infrastructure, such as the turf management of the course, location accessibility, surrounding scenery, and the safety of amenities, is the most crucial antecedent environmental factor that is associated with the cognitive evaluation and emotional satisfaction of older adult participants. This finding supports the core argument of previous studies in sports management and service marketing (17, 19), which posited that excellent environmental stimuli in general commercial fitness centers or large-scale outdoor sports tourism facilities immediately induce a pleasant emotional experience for customers, resulting in strong overall satisfaction and a firmly established favorable brand image. Particularly for older adults, due to the limitations of cognitive and physical functional decline caused by aging, they are inevitably much more sensitive than younger adults to the comfort of the activity environment and, above all, safety features that prevent falls (e.g., flat course designs, highly visible signs, and convenient rest areas). Therefore, it can be interpreted that the qualitative level of a park golf course's servicescape plays a highly dominant and primary role in forming psychological attachment (image) to the facility and practical satisfaction with the service.

Second, it was firmly confirmed that perceived image and satisfaction, which are the individual's internal organismic responses, had statistically significant positive effects on the continuous motivation to participate in exercise, corresponding to the behavioral response (*β* = .447, *β*  = .299). This means that beyond simply experiencing excellent environmental stimuli, older adults who internalize a positive and familiar psychological image of the park golf course and are deeply satisfied with the provided programs and operations exhibit a much stronger intrinsic and extrinsic motivation to participate in the sport. Viewed from the theoretical perspective of the S-O-R paradigm, this is an exemplary causal case in which the cognitive internal state formed by environmental stimuli smoothly and successfully transitions into a specific behavioral and psychological orientation toward the activity. This result is consistent with findings from several previous studies (21, 25) indicating that the positive image and experiential satisfaction strongly perceived by sports tourists or users of large leisure facilities act as a detonator that most powerfully induces psychological immersion, solid revisit intentions, and intrinsic motivation for the activity. An academically impressive and noteworthy point here is the fact that the magnitude of the effect of perceived “image” on motivation (*β* = .447) was statistically much larger and more dominant than the effect of simple usage “satisfaction” (*β* = .299). This strongly suggests that, given the unique cultural characteristics of park golf—where older adults enjoy socializing and leisure over a long period centered around clubs or local senior communities—the symbolic and warm emotional “comprehensive image” the park golf course holds within the local community serves as a much more effective and fundamental mechanism in stimulating and maintaining older adults' continuous sports participation motivation than the short-term, functional “one-time usage satisfaction” provided by the facility.

Third, participation motivation, established as the core response mediator in this study, was shown to have a strong positive effect on the individual's subjective well-being, which is the final outcome variable and ultimate goal (*β* = .774). This substantial explanatory power of the path from motivation to older adults' well-being is one of the most essential and significant academic discoveries revealed by this research model. The data verify that the pure enjoyment (intrinsic participation motivation) acquired in the process of regularly playing park golf to build physical health and stamina, deeply socializing with peers of similar ages, and learning new skills to conquer the course is a decisive and dominant factor that substantially increases the life satisfaction of individuals prone to isolation and maximizes positive affect in daily life. This clear result supports the fundamental premise of motivation theory (11, 38), which posits that human beings fully experience the highest level of psychological well-being when their brain and psychology are activated by voluntarily participating in physical activities based on their own choices (autonomy) and enjoyment (intrinsic motivation) or certain external motivations, rather than external coercion or oppression. Furthermore, it reaffirms and further expands the discussions of recent sports science studies (10, 14)—which reported that positive motivation manifested during sports participation is the strongest predictor for dramatically improving an individual's positive affect and overall quality of life—within the context of park golf, a unique “sport tailored for older adults” in South Korea. In a similar cultural context, a recent multi-center study of Iranian older adults reported that a healthier lifestyle was positively associated with greater motivation for physical activity [OR = 1.18, 95% CI (1.08, 1.29); 39], which provides comparative, cross-cultural support for the central role of motivation in promoting physical activity and well-being among older populations observed in the present study. The active act of gripping a club and walking in a park autonomously, purely for maintaining one's own health and expanding positive social interactions, rather than through external pressure like a family's urging or a doctor's warning, acts as a robust psychological vaccine that helps offset the depression frequently encountered in old age and may promote “successful aging”.

Fourth, the results of the multiple mediation effect analysis utilizing the phantom variable technique within the structural equation model revealed that the servicescape has a mechanism of indirectly affecting the dependent variable, participation motivation, through the cognitive processes of “image” and “satisfaction” perceived by older adults, rather than directly affecting it. The statistical significance of both mediating pathways was supported, which is a decisive point that most empirically demonstrates the theoretical validity and sequential causality of the S-O-R structure through actual field data collected. The essential meaning of this phenomenon suggests that no matter how exceptionally a local government invests massive budgets to create world-class course management, landscaping, and location conditions for a park golf course, if it is not imprinted in the minds of the actual users—older adults—as a positive emotional and functional image of being “safe and friendly,” or if it is not internalized as a definitive, tangible satisfaction with the service due to an inconvenient operating system, it ultimately cannot result in any voluntary and passionate sports motivation. From the perspectives of environmental psychology and consumer leisure behavior, this result holds deep and profound academic significance in that it clearly decodes the complex, causal black-box mechanism of how inanimate physical environmental resource elements accurately penetrate human subtle psychological attitude variables to transition into final motivation and active behavior. Through the rigorous proof of specific mediation effects using phantom variables, this study overcomes the chronic methodological limitations of previous park golf studies that remained at unidimensional relationships and elevates the scientific depth of applying S-O-R theory to the next level.

Based on the extensive academic analysis results and empirical insights derived from this study, highly specific and practical suggestions are proposed to on-site operations managers of the rapidly increasing park golf facilities and to local government policymakers for older adults' welfare to maximize service field application. First, continuous, systematic, and bold budget investments in the physical environment (servicescape) infrastructure of park golf courses are required as a top priority. As the analysis results indicated that the servicescape was the absolute starting point governing older adults' cognitive evaluations and subsequent behaviors, it is imperative to radically improve intuitively tangible environmental qualities. This includes thorough, green turf management, the expansion of large-scale rest areas and comfortable shading facilities to prepare for the summer heat, and the installation of gentle walkways and safety fences to prevent falls, carefully considering the physical characteristics of older adults.

Second, beyond merely emphasizing “functional usage satisfaction” in park golf course operations and local government promotional marketing, a highly sophisticated branding strategy that intensively enhances a strong, “emotional image” is desperately needed. Considering that the analysis results showed symbolic image had a statistically much larger impact on inducing individuals' exercise motivation than functional satisfaction, an emotion-oriented marketing campaign would be more effective than ever. Such a campaign should position the park golf course not simply as a place to hit balls, but as the safest and most vibrant community hub where older adults of similar ages build friendships and share warm interactions and affection.

Third, a meticulous program design that can maximize motivation in the field, rather than controlled extrinsic motivation, must be accompanied. Based on Self-Determination Theory (SDT), policies should actively support the lifelong maintenance of participants' participation motivation without it waning. This involves allowing participants to flexibly choose their own course and rounding intensity according to their stamina and health conditions to support autonomy; hosting mini-tournaments by age group or skill level within the region to confer a sense of achievement and medals to support competence; and actively linking clubhouse social events where participants can drink tea and chat with their companions after finishing a round to support relatedness.

## 5 Limitation and future direction

Despite the contributions of this study, which brought about the convergence of park golf and environmental psychology, several academic and methodological suggestions are left for future researchers to supplement limitations in the exploratory design and further expand the academic depth of this field. First, since this study statistically inferred causal relationships between variables based on cross-sectional survey data collected as a one-time event at a specific point in time, there is a somewhat strict limitation to directly interpreting the long-term causal flow. Future studies must comprehensively adopt a longitudinal panel study design to track and observe in real-time how the perception of the physical servicescape and the subjective well-being of older adults who have started park golf for the first time dynamically change and mature over a long period, such as 6 months, 1 year, or 3 years.

Second, scientific supplementation of the measurement methodology for the constructs is strongly required. This study relied entirely on self-report questionnaires to measure the concepts of psychological motivation and well-being. In subsequent convergent studies, the objective validity of humanities and social sciences research should be further advanced by cross-measuring “physiological medical indicators,” such as a decrease in salivary cortisol hormone levels or blood pressure stabilization, or by concurrently measuring “actual objective physical activity level” data using smartwatches to prove the degree of improvement in subjective well-being.

Third, this study did not measure or control for several individual-level confounders—such as health status, income, and social support—that may influence older adults' motivation and well-being. Because these variables were not collected in the on-site survey, their potential confounding effects could not be statistically controlled, and this should be considered when interpreting the estimated relationships. Furthermore, in terms of research model design, a multidimensional expansion of the analytical model exploring latent moderating variables or sample heterogeneity is absolutely necessary. Even within the same older adult population, the magnitude of sensitivity with which physical environmental stimuli transition into satisfaction or exercise motivation can differ significantly depending on the individual's underlying health conditions, economic income level, prior sports-related knowledge, or the presence of social support from surrounding family members. Therefore, there is a strong academic demand for model expansions that carefully segment older adults' population through Latent Profile Analysis (LPA) or examine the interaction effects of such diverse moderators from various angles.

Lastly, international comparative studies that deeply consider diverse cultural contexts are urgently needed. Park golf, which geographically originated in Hokkaido, Japan, has developed dazzlingly by newly forming a unique and tight-knit club community culture in South Korea, where a distinctive collectivist culture is intermingled. Therefore, if broad cross-cultural studies are conducted by sampling large numbers of older adults in Asian countries like South Korea and Japan, as well as Western countries where park golf is just entering the introductory stage, it will greatly contribute to uncovering the unique cross-national cultural differences in how the Eastern-specific “relatedness”-centered thinking or social face norms affect park golf leisure participation and the formation of psychological well-being.

## 6 Conclusion

This study comprehensively and scientifically investigated the multidimensional and structural relationships among the physical environment of the servicescape experienced on-site, perceived facility image, service participation satisfaction, voluntary exercise motivation, and the ultimate subjective well-being of life. This was conducted among adults actively participating in park golf, which has rapidly emerged as the most representative and popular lifetime sports leisure activity for older adults at a time when society is on the verge of entering a super-aged era, based on the S-O-R paradigm and Self-Determination Theory (SDT). The core conclusions derived through rigorous statistical analysis are as follows.

First, the rational and pleasant servicescape of the park golf course had a very strong, dominant, and positive effect on the positive image and overall satisfaction perceived by older adult participants regarding the facility. Second, the positive image firmly formed in the mind and high tangible satisfaction through an excellent physical environment were shown to significantly increase the sports participation motivation that immerses older adults in park golf activities. Third, it was confirmed that the strongly and voluntarily formed motivation for sports participation is ultimately the most decisive psychological core variable that dramatically enhances the subjective well-being (improved life satisfaction and maximization of positive affect) of older adults in their daily lives. In addition, through the multiple indirect effect analysis pioneering the use of phantom variables, this study demonstrated the unique multiple mediation mechanism that, beyond simply creating an excellent physical environment, it must pass through the cognitive filters of the participants' “image” and “satisfaction” before it can finally transition into a powerful participation motivation.

In conclusion, this study clearly confirmed with empirical data that investing in an excellent physical servicescape environment for park golf courses goes far beyond providing a unidimensional exercise space for physical improvement. By successfully inducing positive cognitive and emotional evaluations from older adults, it strongly imparts a voluntary internal motivation toward a healthy life, ultimately acting as the fundamental and most effective catalyst for drawing out “successful aging” and genuine psychological well-being, which are the main topics of modern aging societies.

## Data Availability

The raw data supporting the conclusions of this article will be made available by the authors, without undue reservation.
